# Expression of SPIG1 Reveals Development of a Retinal Ganglion Cell Subtype Projecting to the Medial Terminal Nucleus in the Mouse

**DOI:** 10.1371/journal.pone.0001533

**Published:** 2008-02-06

**Authors:** Keisuke Yonehara, Takafumi Shintani, Ryoko Suzuki, Hiraki Sakuta, Yasushi Takeuchi, Kayo Nakamura-Yonehara, Masaharu Noda

**Affiliations:** 1 Division of Molecular Neurobiology, National Institute for Basic Biology, Okazaki, Japan; 2 School of Life Science, The Graduate University for Advanced Studies, Okazaki, Japan; University of Maryland, United States of America

## Abstract

Visual information is transmitted to the brain by roughly a dozen distinct types of retinal ganglion cells (RGCs) defined by a characteristic morphology, physiology, and central projections. However, our understanding about how these parallel pathways develop is still in its infancy, because few molecular markers corresponding to individual RGC types are available. Previously, we reported a secretory protein, *SPIG1* (clone name; D/Bsp120I #1), preferentially expressed in the dorsal region in the developing chick retina. Here, we generated knock-in mice to visualize *SPIG1*-expressing cells with green fluorescent protein. We found that the mouse retina is subdivided into two distinct domains for *SPIG1* expression and *SPIG1* effectively marks a unique subtype of the retinal ganglion cells during the neonatal period. *SPIG1*-positive RGCs in the dorsotemporal domain project to the dorsal lateral geniculate nucleus (dLGN), superior colliculus, and accessory optic system (AOS). In contrast, in the remaining region, here named the pan-ventronasal domain, *SPIG1*-positive cells form a regular mosaic and project exclusively to the medial terminal nucleus (MTN) of the AOS that mediates the optokinetic nystagmus as early as P1. Their dendrites costratify with ON cholinergic amacrine strata in the inner plexiform layer as early as P3. These findings suggest that these *SPIG1*-positive cells are the ON direction selective ganglion cells (DSGCs). Moreover, the MTN-projecting cells in the pan-ventronasal domain are apparently composed of two distinct but interdependent regular mosaics depending on the presence or absence of *SPIG1*, indicating that they comprise two functionally distinct subtypes of the ON DSGCs. The formation of the regular mosaic appears to be commenced at the end of the prenatal stage and completed through the peak period of the cell death at P6. SPIG1 will thus serve as a useful molecular marker for future studies on the development and function of ON DSGCs.

## Introduction

Retinal ganglion cells (RGCs) are classified into at least 10–15 types based on morphology and/or physiology in mammalian species [1]–[3]. Each type codes for different features of vision such as motion, color, and contrast, and carries them in parallel into different subcortical areas [4]–[6]. Respective types share the following key characters to exhibit functional specificity: (i) they form regular mosaics individually to cover the retinal surface effectively [7], [8]; (ii) they form synapses only with appropriate presynaptic bipolar and amacrine cells in a distinct sublamina within the inner plexiform layer (IPL) [9], [10]; and (iii) they individually project to a unique region within the brain [11]. However, little is known about how distinct RGC types are generated or how they develop these key features. Because of a lack of molecular markers to selectively label a specific type of RGC early enough in development, investigators had to infer what happened during the mosaic formation of RGCs by examining older tissues [12], [13].

Past studies on mammals suggested that dendritic segregation into ON and OFF sublayers in the IPL is the result of selective elimination of branches by an activity-dependent mechanism following an initial stage of overgrowth [14]–[18]. However, subtypes cannot be readily distinguished by their morphology early in development. On the other hand, time-lapse imaging of zebrafish RGCs demonstrated that most RGCs precisely target synaptic strata within the IPL while each RGC subtype exhibits individual dendritic growth and arborization patterns. This implies that amacrine cells provide stratification cues for their RGC partners [19]. To assess whether this mechanism of stratification is also applicable to some subtypes of the RGCs in mammals, molecular markers are required for distinguishing among the different types of RGCs.

Two types of RGCs are known to code the direction of image motion: the ON-OFF direction selective ganglion cells (DSGCs) and the ON DSGCs [20], [21]. Both these types respond with many spikes to motion in a particular direction and with few spikes to the opposite motion [22]. The ON DSGCs comprise three physiological subtypes distinguished by their preferred directions, corresponding roughly to upward, downward, and temporal to nasal in the visual field [23]. Each physiological subtype is assumed to connect with a selective subset of cholinergic amacrine cells and other presynaptic neurons to achieve the specificity of preferred direction [24]; however, how each subtype is generated and these circuits are organized during development remains totally unknown [25]. It has been assumed that the ON DSGCs provide the sole retinal input to the terminal nuclei of the accessory optic system (AOS), where directional signals from ON DSGCs underlie the optokinetic nystagmus that stabilizes an image on the retina in the presence of head movements [26]. The neurons in the medial terminal nucleus (MTN), a principal terminal nucleus of the AOS, respond best to either upward or downward movement [27]. It has been puzzling that the distribution of MTN-projecting cells is almost random, because other types of RGCs usually form a regular mosaic with a clear exclusion zone [28], [29].

Previously, we described the dorsal-rich expression of a clone, tentatively named D/Bsp120I #1, in the developing chick retina [30]. D/Bsp120I #1 is a secretory molecule of unknown function, which is composed of a follistatin (FS)-like domain, an extracellular calcium-binding (EC) domain, and two immunoglobulin-like domains. Here we refer to this molecule as SPIG1 after SPARC (secreted protein acidic and rich in cysteine: [31])-related protein containing immunoglobulin domains. In the present study, we found that SPIG1-positive ganglion cells in the neonatal mouse retina form a regular mosaic in the ganglion cell layer (GCL) broadly in the ventronasal region, suggesting that they represent a single type of neuron. To characterize this type of RGC, we visualized *SPIG1*-expressing neurons by using genetically encoded *green fluorescent protein* (*gfp*) and examined their spatial organization, dendritic arborization, and axonal projection during the neonatal period. Our study revealed that the MTN-projecting cells form a superimposition of two distinct regular mosaics, and that SPIG1 marks a subtype of the ON DSGCs projecting to the MTN during development. Moreover, monitoring the GFP-labeled RGCs provided new insights into the developmental changes in dendritic stratification and mosaic formation.

## Results

### Expression of *SPIG1* mRNA in the developing mouse retina is asymmetric along the dorsotemporal to ventronasal axis

**Figure 1:**
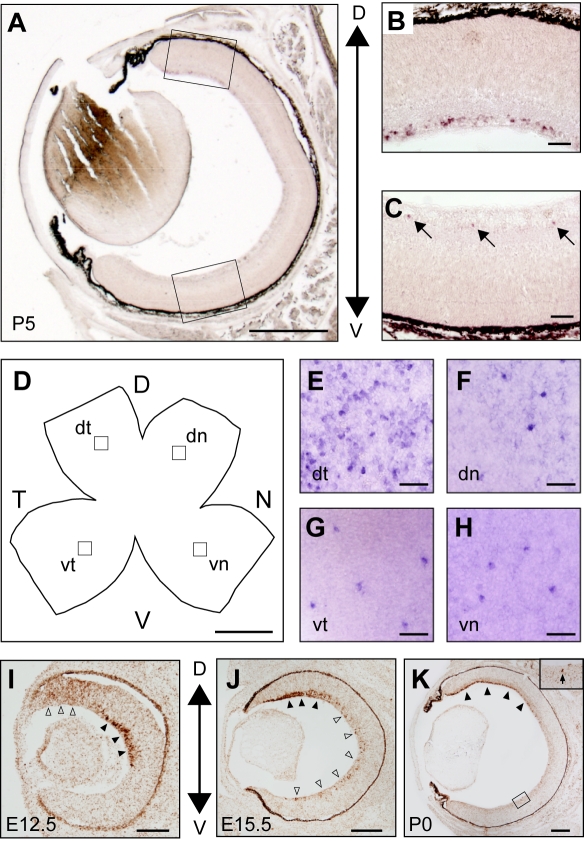
Asymmetric expression of *SPIG1* mRNA in the mouse retina. A–H, Section (A–C) and whole-mount (D–H) *in situ* hybridization with digoxigenin-labeled antisense riboprobes at P5. (B) and (C) are enlargements of the dorsal and ventral regions boxed in (A), respectively. In the dorsal area (B), *SPIG1* is expressed in a large population of cells in the GCL. In the ventral area (C), *SPIG1* is expressed in a small subset of cells in the GCL (arrows). In section *in situ* hybridization, dorsal is upwards, and ventral is downwards. D, A schematic drawing of a flat-mount retina for *in situ* hybridization. D, dorsal; N, nasal; T, temporal; V, ventral. (E–H) are enlargements of the GCL of the dorsotemporal (dt), dorsonasal (dn), ventrotemporal (vt), and ventronasal (vn) areas boxed in (D), respectively. In the dt area (E), *SPIG1*-expressing cells are densely distributed. In the dn, vt, and vn areas (F–H), *SPIG1*-expressing cells form a regular mosaic, indicating that they represent a single type of neuron. I–K, Section *in situ* hybridization of the retinas using a tyramide signal amplification system with diaminobenzidine. At E12.5 (I), the expression is first detected in the outer layer of the dorsocentral retina (black arrowheads) and the entire layer of the dorsoperipheral retina (white arrowheads). At E15.5 (J), the expression peak in the outer layer is shifted to the peripheral region in the dorsal retina (black arrowheads). The expression in the GCL of the ventral retina is first detected in a small subset of cells at this stage (white arrowheads). The expression pattern at P0 (K) is similar to that at P5 (A–C). In the ventral area (K), *SPIG1* is expressed in a small subset of cells in the GCL (arrow in inset). Scale bars: 500 µm (A); 50 µm (B, C, and E–H); 1 mm (D); 100 µm (I); 200 µm (J, K).

We previously identified a catalog of topographic molecules through large-scale screening in the developing chick retina. *SPIG1* (formerly D/Bsp120I #1; GenBank accession number, AF257353 and EF692644) was identified as being preferentially expressed in the GCL in the dorsal region [30]. To examine the *SPIG1* mRNA expression in the developing mouse retina, we performed *in situ* hybridization on coronal sections at P5 (Figure 1A). Consistent with the results in the chick, *SPIG1* was densely expressed in a large population of RGCs in the dorsal retina (Figure 1B). In contrast, in the ventral retina, *SPIG1* was sparsely expressed in a small population of RGCs (Figure 1C). To examine the spatial organization of the *SPIG1*-positive cells in the GCL, we performed whole-mount *in situ* hybridization on the flat-mount retina at P5 (Figure 1D–H). The expression pattern of *SPIG1* in the GCL was markedly asymmetric. In the dorsotemporal region of the retina, *SPIG1*-positive cells were distributed densely (Figure 1E). In the other regions, however, they were only sparsely distributed; they were almost evenly spaced forming a regular mosaic with a clear exclusion zone (∼50 µm; see below) where neighboring *SPIG1*-positive cells were apparently absent from the nearby position in the GCL (Figure 1F–H). Because the distribution in a regular mosaic is a strong indicator of a specific neuronal type in the retina [5], [32], we assumed that *SPIG1* is expressed in a specific type of neuron.

Next we examined the developmental expression of *SPIG1* in coronal sections from E11.5 to P0 by *in situ* hybridization. The expression was first detected at E12.5 in the outer layer of the dorsocentral retina, presumably in the RGC, the earliest generated neurons in the retina (Figure 1I). In addition, the signal was detected throughout the layer in the peripheral region of the dorsal retina (Figure 1I). Between E12.5 and E15.5, the strongly *SPIG1*-positive RGC domain shifted to more dorsal region of the retina (Figure 1J). At E15.5, *SPIG1* was expressed in a large population of RGCs in the dorsal retina and in a small population of RGCs in the ventral retina as at P0 and P5 (Figure 1K and 1C, respectively).

### Targeted expression of GFP from the *SPIG1* gene locus

**Figure 2:**
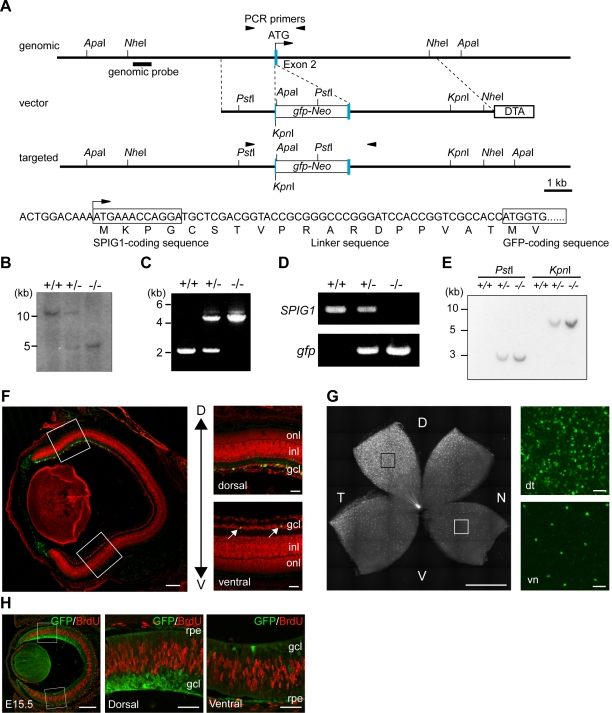
Generation of knock-in mice for the *SPIG1* gene and expression of GFP in the retina. A, Schematic representation of the structure of the endogenous allele (genomic), targeting vector (vector), and targeted allele (targeted). The genomic sequence of the head of exon 2 and the corresponding amino acid sequence in the targeted allele are shown below. The first coding exon of *SPIG1* is indicated by blue boxes. For construction of the targeting vector, a *gfp*-*Neo* cassette was inserted in frame in the signal sequence of *SPIG1* after the first four amino acids, Met-Lys-Pro-Gly, to yield fusion to the N-terminus of GFP through the linker sequence. The DT-A cassette was placed at the 3′ terminus of the homologous region for negative selection. The region used as a probe for Southern blotting is indicated by a bold bar. The position of primer sequences used for PCR analysis is also shown by arrowheads. B, Southern blot analysis of *Apa*I-digested genomic DNA from wild-type (+/+), heterozygous (+/−), and homozygous (−/−) mice. C, Genomic PCR analysis of wild-type (+/+), heterozygous (+/−), and homozygous (−/−) mice. D, RT-PCR analysis of total RNA from the brain of wild-type (+/+), heterozygous (+/−), and homozygous (−/−) mice. E, Southern blot analysis of *Pst*I- or *Kpn*I-digested genomic DNA from wild-type (+/+), heterozygous (+/−), and homozygous (−/−) mice with a probe for the coding region of *gfp*. F, Expression of GFP in the retina of *SPIG1^gfp/+^* mice in a cross section at P5. Enlargements of the boxed regions are shown on the right. In the ventral area, GFP is expressed only in a small subset of cells in the GCL (arrows). In a cross section, dorsal (D) is upwards, and ventral (V) is downwards. inl, inner nuclear layer; onl, outer nuclear layer. G, Expression of GFP in the retina of *SPIG1^gfp/+^* mice in a flat-mount at P5. The retina is apparently subdivided into two domains with *SPIG1* expression, the dorsotemporal and pan-ventronasal domains. Enlargements of the boxed regions are shown on the right. d, dorsal; n, nasal; t, temporal; v, ventral. In the pan-ventronasal domain, *SPIG1*-expressing cells form a regular mosaic. H, Double staining of GFP and BrdU in a cross section at E15.5. Enlargements of the boxed regions are shown on the right. GFP is not expressed in the proliferating cells at S phase. rpe, retinal pigment epithelium. Scale bars: 200 µm (left panels in F/H); 50 µm (enlargements in F/G, and H); 1 mm (left panel in G).

To characterize the *SPIG1*-positive cells, we inserted *gfp* into the *SPIG1* gene through homologous recombination in ES cells (Figure 2A) and visualized the *SPIG1*-expressing cells with the genetically encoded *gfp*. The faithful gene replacement was confirmed by genomic Southern hybridization (Figure 2B) and PCR analysis (Figure 2C). RT-PCR analysis showed that *gfp* transcripts are expressed in place of *SPIG1* transcripts from the targeted allele (Figure 2D). Southern blot analysis with a probe for a coding region of *gfp* confirmed that the targeting vector is not inserted into an ectopic genomic locus (Figure 2E). Heterozygous (*SPIG1^gfp/+^*) and homozygous (*SPIG1^gfp/gfp^*) targeted mice were healthy, fertile, and apparently normal. The genotypic analysis of 477 neonatal offspring obtained from breeding heterozygous animals showed an approximate Mendelian ratio between wild-type (26.8%, n = 128), heterozygous mutant (50.7%, n = 242), and homozygous mutant (22.4%, n = 107) animals.

### The retina is composed of two distinct domains for *SPIG1* expression

The expression of GFP in the retina of *SPIG1^gfp/+^* mice faithfully reproduced the expression of *SPIG1* mRNA in the wild-type mice (compare Figure 1A with 2F, and Figure 1J with 2H); GFP-positive GCL cells were crowded in the dorsotemporal retina, but dispersed in the ventronasal retina at P5. The expression of *gfp* mRNA in the retina of *SPIG1^gfp/+^* mice also faithfully reproduced the expression of *SPIG1* mRNA in the wild-type mice (data not shown). The distribution in the flat-mount retina clearly showed that the retina is subdivided into two domains with respect to *SPIG1* expression along the dorsotemporal to ventronasal axis: the dorsotemporal domain and the broad pan-ventronasal domain (Figure 2G). GFP-positive cells were densely distributed in the dorsotemporal fourth of the retina peaking at the periphery. In the complementary region, here termed the pan-ventronasal domain, GFP-positive cells formed a regular mosaic with a clear exclusion zone.

To confirm that only postmitotic cells express SPIG1, E15.5 embryos of *SPIG1^gfp/+^* mice were given a single injection of BrdU and analyzed 1 h later. Double staining using antibodies against BrdU and GFP showed that BrdU-labeled cells were located in the intermediate zone of the retina and SPIG1-positive cells were present in the nascent RGC layer in the outer retina and devoid of BrdU (Figure 2H). The retinal laminar structure and the distribution pattern of GFP-positive cells in *SPIG1^gfp/gfp^* mice were apparently normal (data not shown).

**Figure 3:**
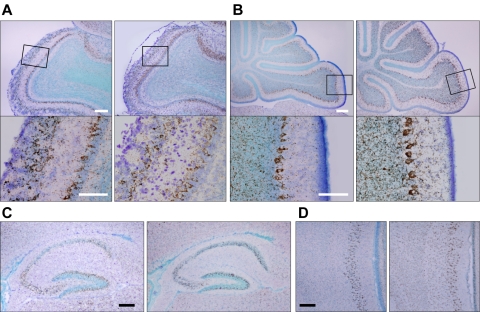
*GFP* mRNA expression in the *SPIG1^gfp/+^* brain faithfully reproduces the expression of *SPIG1* mRNA. *In situ* hybridization for parasagittal sections of P10 brain. The signals were detected by a tyramide signal amplification system. Positive cells were stained brown with diaminobenzidine. Sections were counterstained with toluidine blue. Left and right panels in (A–D) show *SPIG1* and *GFP* expression, respectively. A, Olfactory bulb. *SPIG1* and *GFP* are expressed in the glomerular layer and mitral cell layer. Lower panels are enlargements of the boxed area. B, Cerebellum. *SPIG1* and *GFP* are expressed in the purkinje cells. Lower panels are enlargements of the boxed area. C, Hippocampus. *SPIG1* and *GFP* are expressed in the CA region and granular cell layer of the dentate gyrus. D, Entorhinal cortex. *SPIG1* and *GFP* are expressed in layer II. Layer I is shown to the right side. Scale bars: 200 µm (upper panels in A*/*B; C, D); 100 µm (lower panels in A*/*B).

*SPIG1* mRNA and *gfp* mRNA levels were explored by *in situ* hybridization of P10 brain sections in the wild-type mice and heterozygous mice (Figure 3). The expression was prominent in the glomerular layer and mitral cell layer of the olfactory bulb (Figure 3A), purkinje cells in the cerebellum (Figure 3B), the CA region of the hippocampus, the granular cell layer of the dentate gyrus (Figure 3C), and layer II of the entorhinal cortex (Figure 3D). In addition, the expression was observed in other regions of the brain including the cortex, colliculus, hypothalamus, and medulla oblongata (data not shown). GFP expression in the heterozygous mice thus faithfully reproduced the expression of *SPIG1*; the pattern of GFP expression in the homozygous mutants was macroscopically identical to that in the heterozygous mutants except for the intensity of the expression (data not shown).

Expression of GFP in non-neuronal tissues of *SPIG1^gfp/+^* mice was also explored by anti-GFP immunostaining to intensify the signal at P14 and P70 (only for the testis and ovary). The expression was observed in the heart (myocardium), lung (alveolar cells and smooth muscle cells in the blood vessels), stomach (epithelial cells and cells in the muscle layer), intestine (epithelial cells located at the tip of intestinal villi and cells in the muscle layer), and ovary (oocyte), but not detected in the liver, spleen, kidney, skeletal muscle, or testis (data not shown).

### SPIG1-positive cells in the pan-ventronasal domain are likely ON DSGCs

**Figure 4:**
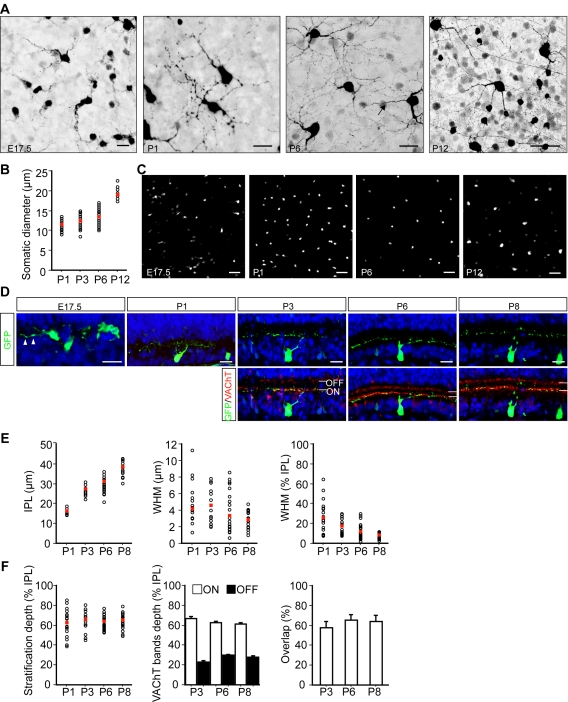
Developmental changes in the morphology and dendritic stratification of GFP-positive RGCs in the *SPIG1^gfp/+^* mouse. A, Z-stack confocal images of GFP-positive RGCs in the flat-mount ventronasal retina at E17.5, P1, P6, and P12. The GFP signals were intensified using an antibody against GFP, and are shown as the inverted gray-scale images. An axon coursing toward the optic fiber layer is visible (arrow). B, Somatic diameter for each GFP-positive ganglion cell plotted against age. The mean values at each age are shown as red squares. The number of samples: 33 at P1; 35 at P3; 40 at P6; and 23 at P12. C, Developmental changes in the mosaic of the GFP-positive cells in the GCL in the flat-mount ventronasal retina. D, Relationships between the distribution of GFP-labeled dendrites (green) and the ON and OFF cholinergic amacrine strata (red) visualized by immunolabeling with anti-VAChT in the pan-ventronasal domain in cross sections. IPL boundaries were determined by using the nuclear stain TO-PRO-3 (blue) with the GCL (below) and the INL (above). At E17.5, processes extending from the GFP-positive cells are observed within the IPL (arrowheads). At P1, dendrites are broadly distributed within the IPL. After P3, the dendrites of the GFP-positive cells predominantly costratify with the ON cholinergic strata. E, The thickness of the IPL plotted against age (left panel). Width at half maximum height (WHM) for each of the GFP-positive RGCs was plotted against age (middle panel). WHM plotted as a percentage of the vertical thickness of the IPL (right panel). The number of samples for all panels: 22 at P1; 17 at P3; 33 at P6; and 21 at P8. F, Dendrite stratification depth of GFP-positive RGCs within the IPL plotted against age. The stratification depth was defined as 0–100% from the INL side to the GCL side of the IPL (left panel). Stratification depth of ON and OFF cholinergic amacrine processes within the IPL (middle panel). The degree of overlap of WHMs of the RGC dendrites and VAChT-labeled cholinergic amacrine processes shown as a percentage of the WHM of the RGC dendrites (right panel). The number of samples for all panels: 22 at P1; 17 at P3; 33 at P6; and 21 at P8. Scale bars: 20 µm (E17.5-P6 in A; D); 50 µm (P12 in A; C).

To examine the morphology of cells expressing *SPIG1* in the pan-ventronasal domain, we intensified GFP signals of *SPIG1^gfp/+^* mice at P6 using an antibody against GFP (Figure 4A). The labeled cells were apparently RGCs, because they each extended an axon towards the optic fiber layer (Figure 4A, third panel, arrow): To show dendrites clearly, axons coursing in the optic fiber layers are mostly not included in the image stacks shown in Figure 4; however, we confirmed that all GFP-positive cells bear an axon coursing towards the optic disc. The mean somatic diameter of GFP-positive cells in the pan-ventronasal domain at P6 was 13.3±0.3 µm (Figure 4B; n = 40; mean±SE). The morphology of these cells was characterized by extensively developed dendrites with a beaded appearance, and in most cases, branched dichotomously. They covered the retina without leaving apparent gaps (Figure 4A, third panel). Somata were usually located approximately at the center of the dendrites (Figure 4A, third panel).

We examined the developmental changes of spatial distribution, the morphology of GFP-positive cells, and somatic diameter in flat-mount retinas of *SPIG1^gfp/+^* mice from E17.5 to P12 in the pan-ventronasal domain (Figure 4A–C). E17.5 is the stage when RGCs start to outgrow their dendrites and the IPL is first observed [33], [34]. At E17.5, a small population of cells in the GCL was labeled by GFP, however, the expression level of GFP was not uniform between cells; some cells were brightly labeled and some were faintly labeled (Figure 4C, leftmost panel). These GFP-positive cells were sparsely scattered over the GCL with little indication of a regular mosaic pattern at this stage. Magnified views of these cells show that some extend a few thick processes laterally (Figure 4A, leftmost panel). At P1, the level of GFP became fairly uniform between cells (Figure 4C, second panel). The mean somatic diameter was 11.4±0.2 µm (n = 33) (Figure 4B). They extended immature, relatively simple dendrites (Figure 4A, second panel).

At P6, the density of GFP-positive cells was apparently decreased and these cells were organized in a considerably regular mosaic array with a clear exclusion zone (Figure 4C, third panel). At P12, just before eye opening, the somatic diameter increased drastically (18.9±0.3 µm; n = 23) compared to that at P6 (13.3±0.3 µm; n = 40) (Figure 4B). A regular mosaic with increased intercellular distance was observed. At this age, in addition to the RGCs with a large somatic diameter, a small subset of presumptive amacrine cells with a small somatic diameter also begin to express GFP in the GCL and inner nuclear layer (INL) (Figure 4A, rightmost panel; 4C rightmost panel). Soon after this age, RGCs began to decrease their GFP expression. As a result, in adult retinas, the expression of GFP was not detected in RGCs anymore in the pan-ventronasal domain (Figure S1, G–I, M–R). In this study, we characterized the GFP-positive cells in the pan-ventronasal domain during the neonatal stage. The morphology of the GFP-positive cells in the dorsotemporal domain was not determined because they were too densely distributed at all ages examined.

### Dendritic stratification of the SPIG1-positive cells in the IPL

To precisely locate the dendritic arborizations of the GFP-positive RGCs, we examined cross sections during retinal development (Figure 4D). It is known that retinal neurons with processes that stratify in the inner half of the IPL are depolarized by increased illumination (ON), whereas those that stratify in the outer half of the IPL are hyperpolarized (OFF) [35]. Cholinergic amacrine cells have terminal processes that stratify in both the ON and OFF sublayers of the IPL [36], and the spatial relationship of cholinergic cell processes with the dendrites of some different types of RGCs has been well examined [37], [38]. By immunolabeling the ON and OFF cholinergic sublaminas with an antibody to vesicular acetylcholine transporter (VAChT), we quantified and compared the vertical distribution of both the GFP-positive ganglion cell dendrites and the cholinergic processes during the period of dendritic remodeling (Figure 4D–F). At E17.5, processes of some GFP-positive cells in the GCL were located in the primitive narrow IPL. At P1, the width of the IPL was increased and defined enough to examine the peak location and dendritic spread within the IPL quantitatively. At P1, dendrites ramified widely across the vertical extent of the IPL, where it became evident that their distribution within the IPL was biased to the inner half. At P3, the dendrites monostratified at the ON sublamina. The monostratification trend became more pronounced at P6 and P8.

To quantify the stratification of dendrites, pixel intensity distribution for GFP as a function of the IPL depth was determined (Figure 4E, F). The vertical spread of dendrites was expressed as the width at half maximum height (WHM). Average WHM values were relatively constant between P1 and P6 (Figure 4E, middle). However, when expressed as a percentage of the IPL, they showed a decrease with age (Figure 4E, right). This may largely reflect an increase in the size of the IPL rather than a decrease in the vertical extent of the dendrites (Figure 4E, left). To determine the extent of overlap between the red (VAChT immunoreactivity of ON sublamina) and green (GFP-positive dendrites) profiles quantitatively, we first calculated the WHM of the red and green peaks for each region [38]. Because VAChT labeling was sparse and two cholinergic bands appeared diffuse at P1, we quantified the vertical distribution of the cholinergic plexuses after P3 (Figure 4F, middle). The dendritic stratification level of the GFP-positive RGCs within the IPL peaked at 65±3% (n = 17; mean±SE) at P3, 63±1% (n = 33) at P6, and 65±2% (n = 21) at P8 (Figure 4F, left). On the other hand, the stratification level of cholinergic amacrine cells peaked at 67±2% (ON) and 23±2% (OFF) (n = 17; mean±SE) at P3, 63±1% (ON) and 30±1% (OFF) (n = 33) at P6, and 61±1% (ON) and 28±1% (OFF) (n = 21) at P8 (Figure 4F, middle). We then measured the overlap of the WHMs of the red and green pixel intensity distribution and expressed this as a percentage of the WHM of the green profile (Figure 4F, right). From P3 to P8, there was significant overlap between the dendrites of GFP-positive cells and the cholinergic plexuses. These results clearly indicate that the dendrites of the GFP-positive RGCs predominantly stratify in the ON cholinergic strata as early as P3. However, dendrites of some GFP-positive RGCs were also found to terminate in the outer half of the IPL at all ages.

Based on morphological properties such as somatic diameter, dendritic branching pattern, and dendritic stratification level within the IPL, we suggest that the GFP-positive RGCs in the pan-ventronasal domain best fit the morphological type Rgc1 of mouse RGCs, which have been identified in retinas of P8, P13, and adult mice [39], [40]. A study making a correlation between the light response property and morphology suggested that ON DSGCs in the mouse retina correspond to the Rgc1 type [41]. In the dorsotemporal retina, dendrites of the SPIG1-positive RGCs were distributed throughout the IPL during the neonatal period suggesting that they are composed of multiple types of RGCs (data not shown).

### Central projection of *SPIG1*-positive RGCs is highly specific

**Figure 5:**
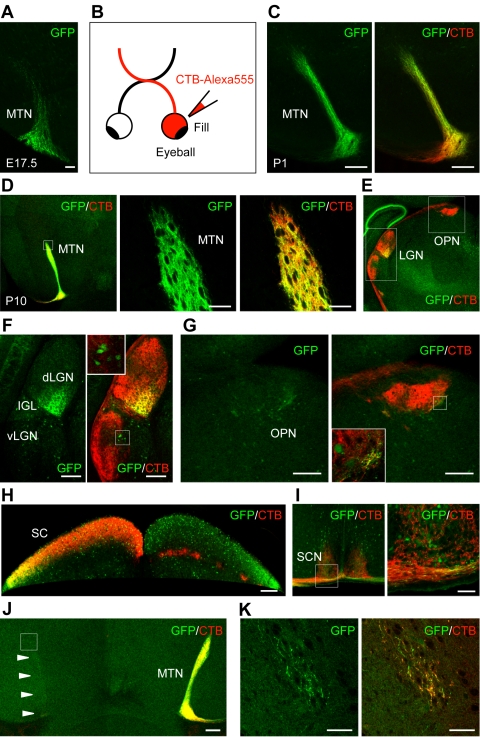
Targets of GFP-positive RGCs in the brain of *SPIG1^gfp/+^* mice. A, Coronal section showing that the MTN begins to be innervated by the GFP-positive fibers at E17.5. B, Schematic drawing of the injection of CTB into the eye. All RGC axons from an eye had been labeled anterogradely by CTB-Alexa 555 one day before the mice were killed at P1 (C) or at P10 (D–K). Except for (H–J), only the contralateral side is shown. C, MTN projection at P1. MTN is already innervated by the GFP-positive retinal fibers extensively. D, MTN projection at P10. The middle and right panels in (D) are enlargements of the dorsal region of the MTN boxed in the left panel. E, Coronal section at the level of the caudal LGN and pretectum. F, Enlargements of the LGN boxed in (E). GFP-positive RGC fibers predominantly innervate the ventro-medial part of the dLGN and are absent from the vLGN and the IGL. Inset is the enlargement of the boxed region in the vLGN, showing that intrinsic GFP-positive neurons are sparsely distributed in this region. G, Enlargements of the OPN boxed in (E). Inset is the enlargement of the boxed region, showing that a small number of GFP-positive RGC fibers innervate the ventromedial part of the OPN. H, Projection to the SC. GFP-positive RGC fibers predominantly innervate the lateral part of the SC. I, Coronal section just posterior to the optic chiasm. The right panel in (I) is the enlargement of the SCN boxed in the left panel, showing that the retinal axon projection to the SCN is devoid of axonal GFP. J, MTN projection in mice enucleated unilaterally at P2, injected with CTB into the remaining eye at P9, and killed at P10. GFP axons contralateral to the enucleated eye selectively disappeared (arrowheads). K, Enlargements of the dorsal region boxed in (J) of the MTN contralateral to the enucleated eye. A small number of GFP-positive RGC fibers remain after enucleation. MTN, medial terminal nucleus; SC, superior colliculus; LGN, lateral geniculate nucleus; dLGN, dorsal LGN; vLGN, ventral LGN; IGL, intergeniculate leaflet; OPN, olivary pretectal nucleus; SCN, suprachiasmatic nucleus. Scale bars: 50 µm (A, D, K); 200 µm (C, F–J).

We found that GFP intensely labels the axonal projections of *SPIG1*-positive RGCs throughout the optic pathway in the *SPIG1^gfp/+^* mice. Because it has been assumed that ON DSGCs project specifically to the AOS, we next assessed whether *SPIG1*-positive RGCs in the pan-ventronasal domain indeed project to the AOS. Analysis of coronal brain slices of SPIG1*^gfp/+^* mice at E17.5 revealed that the MTN, a principal terminal nucleus of the AOS, begins to be innervated by GFP-positive axons (Figure 5A). Next, the retinofugal axons of *SPIG1^gfp/+^* mice at P1 and P10 were examined by labeling them with cholera toxin subunit B (CTB)-Alexa 555 that had been injected into the unilateral eyeball 24 h before (Figure 5B). Strikingly, a large proportion of the CTB-labeled retinal axons terminating in the MTN were GFP-positive at both ages (Figure 5C, D). In other AOS nuclei, the dorsal terminal nucleus (DTN) and the lateral terminal nucleus (LTN), only a small proportion of the innervating axons were GFP-positive (data not shown).

To identify other central targets of the GFP-positive RGCs, we examined sections in detail at P10. Figure 5E shows a coronal section at the level of the caudal LGN and pretectum. Figure 5F and G are enlargements of the lateral geniculate nucleus (LGN) and olivary pretectal nucleus (OPN) boxed in Figure 5E, respectively. Numerous GFP-positive RGC axons terminated in the dorsal LGN (dLGN), with caudal-high and ventromedial-high gradients (Figure 5F). In contrast, the ventral LGN (vLGN) and the intergeniculate leaflet (IGL) were devoid of GFP-positive RGC axons (Figure 5F): In these regions, GFP labeled only the somata and dendrites of the intrinsic SPIG1-potitive cells (Figure 5F inset). In the OPN, only a tiny proportion of the innervating retinal fibers were GFP-positive, and they were confined to the ventromedial region of the nucleus (Figure 5G inset). In the superior colliculus (SC), GFP labeled both afferent fibers of the SPIG1-positive RGCs and the somata and dendrites of the intrinsic SPIG1-positive cells in the superficial layers of the SC (Figure 5H). The projection of GFP-positive RGCs to the SC was confined to the lateral region, in contrast to the uniform distribution of the CTB label in the entire SC. The topographic projections to the SC and dLGN are well correlated with the topographic expression pattern of *SPIG1* in the retina. GFP-positive axons were excluded from the suprachiasmatic nucleus (SCN) (Figure 5I).

In some central targets, it was slightly difficult to distinguish afferent RGC axons and dendrites of intrinsic *SPIG1*-expressing cells. Moreover, we could not exclude the possibility that some of the GFP-positive axons observed in the central targets originated from non-retinal regions. Therefore, to confirm that GFP-positive axons observed in the central targets originated from the retina, we examined P10 *SPIG1^gfp/+^* mice that were enucleated unilaterally at P2 and injected with CTB into the remaining contralateral eyeball at P9. On the side contralateral to the enucleated eye, virtually all of the GFP-positive axons in the MTN disappeared (Figure 5J): Some CTB-labeled axons, presumably originating from the ipsilateral retina, remained after the enucleation and a large proportion of these axons were GFP-positive (Figure 5K). This was also the case for other target nuclei (data not shown). Thus, the enucleation experiments demonstrated that almost all the GFP labeling corresponded to RGC axon termini in the central targets. The highly restricted pattern of central innervation of GFP-positive RGC axons at P10 was essentially same between the heterozygous and homozygous mice (data not shown).

### SPIG1-positive RGCs in the pan-ventronasal domain exclusively project to the MTN

**Figure 6:**
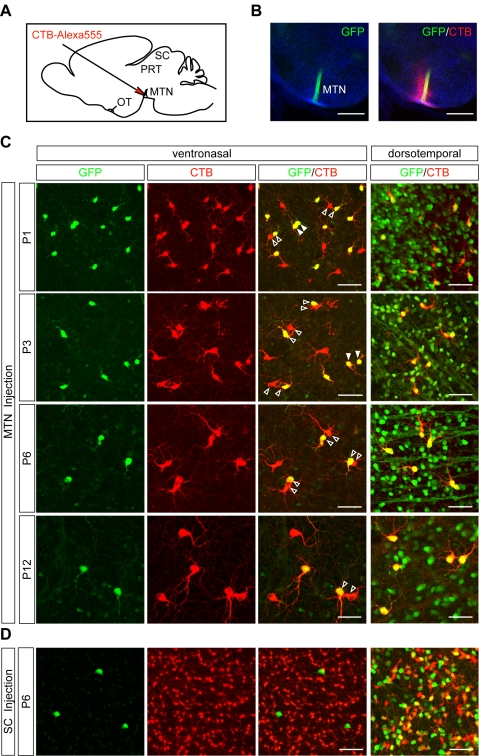
Developmental characterization of SPIG1-positive RGCs projecting to the MTN and SC. CTB-Alexa 555 was injected into the MTN (C) or SC (D) to retrogradely label RGCs. A, Schematic drawing of the injection of CTB into the MTN of neonatal mouse brain (parasagittal view). The glass capillary was inserted at an oblique angle from the vertical. OT, optic tract; PRT, pretectum. B, The injection site in a coronal slice (200 µm thick) at P6, showing that the GFP-positive fibers (green) innervating the MTN are labeled with injected CTB (red). C, Developmental analysis of MTN-projecting RGCs in the neonatal period. Paired cells are a common feature of the MTN-projecting cells (black and white arrowheads in the third column). In the pan-ventronasal domain (left three columns), approximately half of the MTN-projecting cells are marked by GFP at all ages. Strikingly, one of the paired cells is always GFP-positive at P6 and P12 (black arrowheads). However, occasionally at P1 and P3, both of the paired cells were found to be GFP-positive (white arrowheads): Here, the incidence of both paired-cells being GFP-negative was low. In contrast, in the dorsotemporal domain, all of the MTN-projecting cells are marked by GFP at all ages (rightmost column). D, GFP expression in RGCs projecting to the SC. In the pan-ventronasal domain, the SC-projecting cells are devoid of GFP expression. In the dorsotemporal domain, approximately half of the SC-projecting cells are marked by GFP. Scale bars; 500 µm (B); 50 µm (C, D).

Because it became clear that a large proportion of the MTN-projecting axons were GFP-positive, we next performed retrograde tracing experiments with postnatal mice to examine whether the GFP-positive RGCs in the pan-ventronasal domain innervate the MTN. We injected CTB-Alexa 555 unilaterally into the MTN of *SPIG1^gfp/+^* mice 24–48 h before (Figure 6A) and examined the retinas at P1, P3, P6, and P12. MTN of *SPIG1^gfp/+^* mice was easily identified by the GFP signal of innervating retinal fibers in coronal brain slices (Figure 6B). CTB-labeled RGCs were distributed throughout the contralateral retina at all ages (Figure 6C). The total number of MTN-projecting cells in the GCL of a contralateral retina was 1,339±160 at P1, 1,304±88 at P3, 1,027±26 at P6, and 845±25 at P12 (n = 3; mean±SE). Approximately 80% of these cells resided in the pan-ventronasal domain throughout the developmental period. Here a slight regional difference was observed in the density of the MTN-projecting cells; for example at P6, the density was highest in the dorsonasal and ventrotemporal quadrants (∼130 cells/mm^2^ in the mid-peripheral region), moderate in the ventronasal quadrant (∼110 cells/mm^2^), and lowest in the dorsotemporal quadrant (∼85 cells/mm^2^) (Figure S2, A–D).

In the pan-ventronasal domain, almost all of the GFP-positive cells were labeled with CTB (91.8±1.7% at P1; 98.1±0.4% at P3; 94.2±1.2% at P6; 94.5±2.0% at P12; n = 3; mean±SE). Conversely, nearly half of the CTB-labeled cells were GFP-positive (59.5±4.0% at P1; 52.5±3.6% at P3; 47.7±2.2% at P6; 47.8±2.4% at P12; n = 3). Of note, CTB-labeled cells tend to exist in pairs (Figure 6C black arrowheads). At P1 and P3, some pairs of CTB-labeled cells were both positive for GFP (Figure 6C white arrowheads) or both negative for GFP (data not shown). These homotypic cell pairs, occasionally clusters of more than three cells, were no longer found at P6 and P12. After P6, only one of the paired cells was always GFP-positive. The GFP-positive and -negative cells did not seem to differ in morphological features such as branching pattern and stratification. In the dorsotemporal domain, only minor populations of GFP-positive cells were labeled with CTB; conversely, almost all labeled cells were GFP-positive throughout neonatal period (Figure 6C, rightmost panels). We also found that a small number of cells (approximately 10 cells per retina) were retrogradely labeled with CTB from the ipsilateral MTN in all ages examined: Approximately half of them were GFP-positive (data not shown).

Next, we injected CTB into the SC of the *SPIG1^gfp/+^* mice and examined the contralateral retina at P6 and P13 (Figure 6D; Figure S1, D–F). The SC was chosen because the vast majority of RGCs project to it [42], and the MTN-projecting cells are known not to send any collaterals to the SC [28]. In the pan-ventronasal domain, the SC-projecting cells were all GFP-negative. This result is consistent with the view that the GFP-positive cells in the pan-ventronasal domain exclusively innervate the MTN and do not send any collaterals to the SC. In contrast, approximately half of the SC-projecting cells in the dorsotemporal domain were GFP-positive (Figure 6D, rightmost panel).

**Figure 7:**
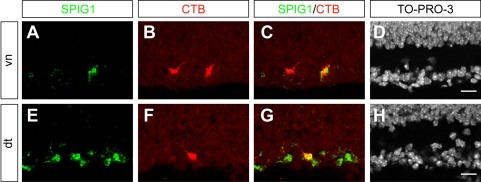
*SPIG1* mRNA expression in the MTN-projecting cells at P6. CTB-Alexa 488 was injected into the MTN of wild-type mice at P5. Fluorescence *in situ* hybridization was performed with the retinal sections labeled retrogradely. Fluorescence of HNPP and CTB-Alexa 488 were pseudocolored as green and red, respectively. A–D, The ventronasal (vn) retina. Approximately half of the MTN-projecting cells are marked by *SPIG1* mRNA expression. E–H, The dorsotemporal (dt) retina. All of the MTN-projecting cells are marked by *SPIG1* mRNA expression. Scale bars; 20 µm (D, H).

To confirm that *SPIG1* mRNA is expressed in half the population of the MTN-projecting cells as demonstrated by GFP, we injected CTB-Alexa 488 into the MTN of wild-type mice (n = 3) at P5. Then we performed fluorescent *in situ* hybridization for *SPIG1* mRNA in retrogradely labeled RGCs in cross sections of the contralateral retina at P6 (Figure 7). In the pan-ventronasal retina, 88.4±1.0% of *SPIG1* mRNA-positive cells (n = 174; mean±SE) were labeled with CTB; conversely, 49.2±1.4% of CTB-labeled RGCs (n = 313) were *SPIG1*-positive (Figure 7A–D). In the dorsotemporal retina, all CTB-labeled RGCs (n = 43) were *SPIG1*-positive (Figure 7E–H). These percentages are almost identical to that for GFP-positive cells, also showing that GFP is faithfully expressed in the *SPIG1*-positive cells in the native retina.

### MTN-projecting cells are subdivided into two distinct regular mosaics

Retrograde tracing experiments (Figure 6) showed that nearly all of the GFP-positive cells in the pan-ventronasal domain project to the MTN from P1 to P12, and these cells constitute about half of the MTN-projecting cells in the domain. Remarkable to us was that GFP-negative cells that project to the MTN also form a regular mosaic; these GFP-negative cells also had their own exclusion zone. To quantitatively verify this notion, we assessed the spatial autocorrelation of GFP^+^·CTB^+^ and GFP^−^·CTB^+^ cells in the pan-ventronasal domain from P1 to P12 by measuring the density recovery profile (DRP) which describes the spatial density of homotypic cells as a function of distance from each other [43]. To begin with, we mapped the position of all the CTB^+^ cells, GFP^+^·CTB^+^ cells, and GFP^−^·CTB^+^ cells in retrogradely labeled retinas (P1-P6, n = 3; P12, n = 2) on the contralateral side. Here CTB^+^ cells represent the MTN-projecting cells.

**Figure 8:**
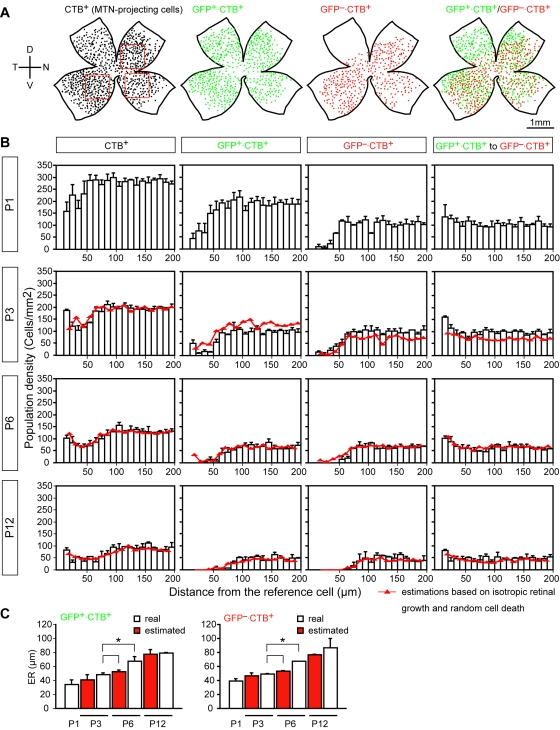
Development of two distinct but interdependent regular mosaics within the MTN-projecting cells. A, Representative reconstructions of the distribution of the MTN-projecting cells in a flat-mount retina at P6. CTB^+^ cells (MTN-projecting cells; black dots) are subdivided into two populations; GFP^+^·CTB^+^ cells (green dots) and GFP^−^·CTB^+^ cells (red dots). Note that all CTB^+ ^cells are marked by GFP in the dorsotemporal domain. Positions quantified for DRPs were exemplified by the squares in the leftmost reconstruction. B, DRPs for retrogradely labeled cells in the pan-ventronasal domain at P1, P3, P6, and P12 (P1-P6, 9 fields from 3 retinas; P12, 6 fields from 2 retinas). The white bars represent the real data, whereas the red lines represent estimated DRP values calculated from the DRP of the respective former developmental ages based on the data of isotropic retinal growth and random cell death. DRPs for CTB^+^ cells (MTN-projecting cells) show that they do not have clear exclusion zones (leftmost column). DRPs for GFP^+^·CTB^+^ cells show that they develop a regular mosaic with an exclusion zone (second column). DRPs for GFP^−^·CTB^+^ cells show that they too develop a regular mosaic with an exclusion zone (third column). Spatial correlations between GFP^+^·CTB^+^ cells and GFP^−^·CTB^+^ cells show that the two different populations tend to exist as pairs with respect to each other at all ages (rightmost column). C, The ER calculated from real (white bars) and estimated (red bars) DRP data for GFP^+^·CTB^+^ cells (left panel) and GFP^−^·CTB^+^ cells (right panel). The significant increases in the ER between the real P3 data and real P6 data are not reproduced with the estimated P6 values. *p<0.05. Values are the mean±SE.

Figure 8A shows drawings of labeled cells in a representative retina at P6, indicating that the CTB^+^ cells in the pan-ventronasal domain are divided into two independent regular mosaics depending on the presence or absence of GFP. DRPs for all MTN-projecting cells in the pan-ventronasal domain show developmental changes in the spatial ordering of the cells from P1 to P12 (Figure 8B leftmost column). At P1, the DRP for near distances showed a reduction, indicating that the possibility that another cell is located nearby becomes lower: The leftmost bar in the DRPs corresponds to the density of tightly neighboring cells, because the somatic diameter of CTB^+^ cells is 12–20 µm. After P3, the DRP for all the CTB^+^ cells shows a trough at around 20–60 µm from the reference cell. This feature was maintained up to P12, although the overall density continues to decrease. The DRP for GFP^+^·CTB^+^ cells (second column) at P1, in contrast, already begins to show mosaic features. At P3, the mosaic features become more apparent although homotypic cell pairs still exist. At P6, homotypic cells at near distance are completely removed and a clear exclusion zone emerges. The radius of the exclusion zone is further increased at P12. The DRP features for GFP^−^·CTB^+^ cells (third column) were similar to those for GFP^+^·CTB^+^ cells at the respective age: Although the plateau density of the GFP^−^·CTB^+^ cells was less than two-thirds of that of GFP^+^·CTB^+^ cells at P1, these two populations showed an almost equal cell density from P3 to P12. The DRPs in the rightmost column represent the spatial crosscorrelation between the GFP^+^·CTB^+^ population and GFP^−^·CTB^+^ population. The DRPs showed a peak at a short distance at all ages, indicating that the two populations with and without GFP expression tend to exist in pairs with respect to the other at all ages examined. Thus, the DRP analysis demonstrated that SPIG1-positive and -negative cells projecting to the MTN form individual but interdependent regular mosaics, and the mosaic regularity for each population appeared to increase continuously after birth.

We found that the number of MTN-projecting cells decreases by 2.6% between P1 and P3, 21.3% between P3 and P6, and 17.7% between P6 and P12. Meanwhile, the retina increased in area by a factor of 1.40 between P1 and P3, 1.17 between P3 and P6, and 1.31 between P6 and P12. We estimated DRP features of the respective ganglion array of the next developmental stages from the former stages based on the assumption that an isotropic expansion occurs by a factor corresponding to the increase in the retinal area, and a decrease in the overall population density of the MTN-projecting cells takes place independently of cell position (Figure 8B, red lines). The assumption of isotropic growth appears to be reasonable, because the plateau values of the DRPs estimated for the CTB^+^ population are very similar to the real data (Figure 8B, leftmost column).

When the actual DRP of GFP^+^·CTB^+^ cells at P3 and the corresponding profile of prospect were compared, the estimated value was lower than the real one tightly nearby but higher at a distance (Figure 8B second column). This indicates that the fraction of GFP^+^·CTB^+^ cells that disappeared between P1 and P3 was larger than estimated, although the cell population tightly close to other homotypic cells was sustained. However, the cells nearby (10–50 µm) were completely removed between P3 and P6. This developmental time window roughly coincides with the peak period (P2–P5) of cell death of RGCs in the mouse retina [44]. At P12, the densities estimated from the DRPs at P6 were very similar to the real values, indicating that the loss of 17.7% of the cells that took place almost randomly between P6 and P12 is the main change during this period. In contrast to GFP^+^·CTB^+^ cells, the population density of GFP^−^·CTB^+^ cells at P3 was larger than the corresponding estimate at a distance (Figure 8B third column), indicating that GFP^−^·CTB^+^ cells are apparently increased between P1 and P3. Between P3 and P6, complete exclusion zones of GFP^−^·CTB^+^ cells seemed to be created by position-dependent cell removal, as well as GFP^+^·CTB^+^ cells. Between P6 and P12, cell loss continuously took place position-independently. On the other hand, the actual population densities in the crosscorrelation were larger than the corresponding values obtained by estimation from P1 for P3 especially at near position (Figure 8B rightmost column), indicating that the density of heterotypic cells nearby increased between P1 and P3. After P6, the estimated densities were very close to the real values, indicating that the cell loss occurred with no relation to the positioning of heterotypic cells.

We then quantified the exclusion zone by calculating the effective radius (ER) [43]. Figure 8C shows the developmental change in the average ER (white bar) obtained for the GFP^+^·CTB^+ ^cells (left panel) and GFP^−^·CTB^+^ cells (right panel), together with the values obtained from the estimation based on the isotropic retinal expansion and position-independent cell loss (red bar). This analysis quantitatively showed that significant increases in the ER for GFP^+^·CTB^+ ^cells (48.1±2.4 µm at P3; 67.3±6.5 µm at P6; mean±SE; p<0.05) and GFP^−^·CTB^+^ cells (49.3±1.0 µm at P3; 67.1±0.6 µm at P6; p<0.05) occurred between P3 and P6, while which were not replicated between the real P3 data and the estimation at P6 (52.1±2.6 µm for GFP^+^·CTB^+ ^cells; 53.3±1.1 µm for GFP^−^·CTB^+^ cells). Thus, significant position-dependent cell loss resulted in the increases in the ER between P3 and P6 for GFP^+^·CTB^+^ and GFP^−^·CTB^+^ mosaics.

In contrast, in the dorsotemporal domain, all of the CTB-labeled cells were GFP-positive at all ages examined (Figure 6C; Figure 8A; Figure S1, J–L). The DRP for the GFP^+^·CTB^+^ population in the dorsotemporal domain at P6 showed no sign of an exclusion zone (Figure S2, E–H). This strongly suggests that the dorsotemporal population is a mixture of cells of different physiological subtypes.

## Discussion

Identifying different functional types of neurons is central to a bottom-up understanding of how networks of neurons give rise to perception and behavior. However, only a few cell-type-specific markers are known at the level of functional cell types in most circuits in the central nervous system [45]. RGCs reportedly comprise more than a dozen types of cells with different dendritic morphological features, but they have not yet been distinguished by specific markers [39], [46]–[48]. Moreover, early in development, they cannot be readily distinguished, because only a mature morphology is generally available for identifying and classifying RGCs. By using genetic labeling, we explored developmental changes in dendritic stratification and mosaic formation of the SPIG1-positive subtypes of ganglion cells during the early neonatal period in the mouse retina. Our study demonstrates that SPIG1 effectively functions as a developmental marker for one of the two functionally distinct subtypes of ON DSGCs exclusively projecting to the MTN in the pan-ventronasal domain.

### MTN-projecting cells are likely ON DSGCs

Our knowledge about the RGCs projecting to the MTN in mice is highly limited, although these cells have been previously investigated in rabbits and rats [28], [49], [50]. We showed that the total number of MTN-projecting cells, including both contralaterally and ipsilaterally projecting cells, is approximately 850 at P12. Assuming that there are 60,000 RGCs in the mouse retina at P12 [51], 850 cells correspond to 1.4% of the total. This number is very close to the 1.5 % reported for an adult rat retina [28], indicating that there is little variability in the density of the MTN-projecting cells between rats and mice. Studies in rabbits suggested that the MTN-projecting cells are morphologically identical to ON DSGCs [50], [52]. In mice, ON DSGCs were identified by electrophysiological experiments; the injection of a dye into the recorded cells revealed that ON DSGCs are cofasciculated with the ON cholinergic plexus labeled by VAChT [41]. They were morphologically classified as type Rgc1 [39], [40]. We found that SPIG1-positive cells best resemble the Rgc1 cells, as judged from somatic diameter, dendritic morphology, and dendritic stratification level within the IPL (Figure 4). Although we characterized SPIG1-positive cells only before eye opening (E17.5-P12) due to the time-dependent expression of SPIG1, it has been reported that most of the RGCs at P8 already exhibit an adult-like dendritic morphology and can be classified into subtypes with the criteria used for adults [40], [53]. Therefore, our characterization of SPIG1-positive RGCs before eye opening would be effective for identification of the RGC type. Notably, dendritic stratification in ON cholinergic strata (Figure 4D) strongly supports our view that SPIG1-positive cells correspond to ON DSGCs projecting to the MTN.

### Distinct regular mosaics revealed by *SPIG1* expression

It has long been puzzling that the MTN-projecting cells do not form a regular mosaic, because morphologically a single type of RGC, the ON DSGC, was assumed to project to the MTN. A recent study of the rat retina pointed out the possibility that the pattern of distribution of MTN-projecting cells involves more than two regular, but spatially independent mosaics that overlay [29]. Using *SPIG1* expression, we revealed for the first time that the MTN-projecting cells in the pan-ventronasal domain of the retina form two distinct regular mosaics overlaying with equal density (Figure 8). Moreover, we demonstrated that these two mosaics are spatially interdependent: cell types of different populations tend to be positioned closely as pairs (Figure 8B). Here, one of the paired cells is SPIG1-positive and the other is SPIG1-negative (Figure 6C). Very recently, it was reported that the closest ON DSGCs, displaying the greatest dendritic overlap, are inclined to show a different directional preference [54]. It is thus probable that the MTN-projecting cells in the pan-ventronasal domain comprise two functionally distinct subtypes of ON DSGCs.

In the MTN, there exist two neuronal populations functionally distinguishable: one is stimulated by upward- and the other by downward-directed motion [27]. These two populations are likely segregated anatomically in the MTN, where neurons in the dorsomedial portion are predominantly stimulated by upward movements and those in the ventrolateral portion by downward movements in rats [55]. As compared with GFP-negative axons, GFP-positive axons are biased to terminate in the dorsal portion of the MTN (Figure 5D, J), suggesting that the *SPIG1*-positive subtype of ON DSGC corresponds to neurons sensitive to upward movements in the retina. Our targeted electrophysiological recordings from the *SPIG1*-positive cells have verified this notion (to be published elsewhere). In the dorsotemporal domain, all of the MTN-projecting cells are marked by SPIG1 from P1 to the adult stage (Figure 6C; Figure S1J–L). The DRP for these cells at P6 shows a peak at 30–40 µm and a trough at around 40–70 µm from the reference cells (Figure S2, A, E). This might indicate that the MTN-projecting cells in the dorsotemporal domain comprise two or more distinct subtypes of ON DSGCs and the heterotypic cells tend to exist 30–40 µm away from each other.

### Dendritic development of SPIG1-positive RGCs

It has been long believed that the initially exuberant dendrites of immature RGCs establish their mature monostratified state through an activity-mediated process requiring the release of glutamate by developing bipolar cells [14]–[18]. However, recent publications argue against the view that glutamate-mediated activity plays important roles in the early refinement of mouse RGC dendrites. Two-photon imaging of the neonatal mouse retina revealed that dendrites of a subset of ganglion cells follow the cholinergic processes as early as P3–P4 [38]. *In vivo* imaging of zebrafish also demonstrated that most RGCs precisely target synaptic strata within the IPL without significant dendritic pruning [19]. Dendrites of RGCs with SPIG1 expression also appear to target the ON cholinergic plexus without significant pruning: Nearly all SPIG1-positive cells are unstratified within the IPL at P1, and become stratified close to ON cholinergic strata by P3 (Figure 4D–F). These stages occur long before bipolar cells form their synaptic contacts, because ribbon synapses between bipolar cells and RGC dendrites have not been established until P10 in the mouse retina [56]–[58]. Our findings thus support the notions that the emergence of an adult-like morphology of RGCs in mice does not require the formation of glutamatergic synapses [40], [53].

### Development of the mosaic in the retina

It has been impossible to directly monitor the development of the ganglion cell mosaic because of the lack of molecular markers for specific RGC types. In this study, we followed developmental changes in the mosaic pattern of two subtypes of RGCs during the neonatal period in the pan-ventronasal domain of the retina. We have shown that both GFP^+^·CTB^+^ cells and GFP^−^·CTB^+^ cells are already organized into a regular mosaic at P1, though neighboring GFP^+^·CTB^+^ cells are infrequently (Figure 4C; Figure 6C; Figure 8); the inclination of the exclusion at a distance of less than ∼50 µm is detectable from the DRP profile in both populations (Figure 8). Because this sign was not observed at E17.5 (Figure S3) in GFP^+^ cells, the exclusion was thought to begin during this period. P1 is a stage when the number of RGCs nearly reaches its peak value [51]. In addition, the MTN-projecting cells decreases only 2.6% between P1 and P3, and the peak period of cell death begins afterward (P2–P5) [44]. This strongly indicates that cell death is not the cause of the beginning of regular mosaics observed at P1. So far, three developmental mechanisms have been hypothesized for the formation of a mosaic by retinal neurons; (i) lateral inhibition [59], [60], (ii) tangential migration [61]–[64], and (iii) cell death [12], [65], [66]. Most of these insights come from studies on cholinergic amacrine cells and horizontal cells, because specific molecular markers only for these cell classes have been available in early development. Here we speculate that the lateral inhibition and/or tangential migration mechanisms is responsible for the formation of the mosaics observed at P1. The action of the Notch pathway is thought to be involved in specific cell-fate decisions via a lateral inhibitory mechanism [59]. In addition, ganglion cells are observed to migrate tangentially as much as 150 µm at this stage in the mouse retina [61], [62]. Therefore, it is possible to speculate that SPIG1-positive and -negative cells migrate tangentially in the GCL to yield a minimal distance between the homotypic cells. The tangential migration is suggested to be driven by short-range repulsive interactions between cells [64]. Here, SPIG1 could be a substance for recognition by the homotypic cells.

We found that the plateau densities for GFP^+^·CTB^+^ and GFP^−^·CTB^+^ cells exhibit a big difference at P1 (∼180 cells/mm^2^ vs. ∼110 cells/mm^2^), but become equal (∼100 cells/mm^2^) at P3 (Figure 8B); during this period the retina increases in area by a factor of 1.40. Surprisingly, the population density of GFP^−^·CTB^+^ cells at P3 indicates that their absolute number is increased during this period. Because the neurogenesis of RGCs ceases by E19 [67], it cannot be explained by novel neurogenesis of SPIG1-negative cells. Here we propose two mechanisms that may explain the deviation of real DRP data of GFP^+^·CTB^+^ and GFP^−^·CTB^+^ cells at P3 from the estimated values. The first conceivable mechanism is the cell-fate change; a fraction of SPIG1-positive cells turned SPIG1-negative by stopping the gene expression. Consistently, heterotypic cell pairs at a distance of less than 30 µm become evident between P1 and P3 (Figure 8B, rightmost panel at P3); this is also recognizable from the DRP profile of CTB^+^ cells at P3 (Figure 8B, leftmost panel). From these observations, we can speculate that GFP^+^·CTB^+^ cells at P1 include the precursor population of GFP^-^·CTB^+ ^cells, and they are further differentiated to establish a new distinct population at P3. However, it is difficult to experimentally examine whether the alteration of SPIG1 expression is directly relevant to the functional alteration of cell types, because the functional difference in MTN-projecting RGCs can be verified electrophysiologically only after P10, when neural connections with amacrine and bipolar cells have been established. The second mechanism concerns the difference in retrograde-labeling efficiency by CTB. It is possible that axons of GFP^−^·CTB^+^ cells do not reach on the MTN fully until P3. If it were the case, only a fraction of GFP^−^·CTB^+^ cells were labeled with CTB at P1, and the density of GFP^−^·CTB^+^ cells at P1 was underestimated.

Our data at P6 clearly demonstrate that closely-positioned homotypic cells are selectively lost and clear exclusion zone is developed between P3 and P6 (Figure 8B, C). It is difficult to consider tangential migration as a mechanism for the formation of an exclusion zone between P3 and P6, because dendrites of SPIG1-positive cells were already monostratified at the ON cholinergic strata at P3 (Figure 4D–F). Moreover, cells during migration were not observed during this period. In line with our view, it has been suggested that a spatially selective loss of ganglion cells is responsible for the increase in regularity of the RGC mosaic pattern [12]. Our results also show that the dendritic stratification of SPIG1-positive cells takes place by P3 (Figure 4D–F) before the elimination of closely-positioned homotypic cells (between P3 and P6) (Figure 8B). This suggests the possibility that dendritic stratification into specific sublaminas of the IPL is a prerequisite for the elimination of neighboring homotypic cells. Dendritic costratification with cholinergic cells indicates that SPIG1-positive cells may receive cholinergic inputs as early as P3. It has been shown that nicotinic acetylcholine receptor-mediated spontaneous retinal waves during the neonatal period are involved in the refinement of retinocollicular projections [68] and the dendritic stratification of RGCs [69]. Therefore, it is worth examining whether acetylcholine receptor-mediated spontaneous retinal waves are necessary for the dendritic stratification and mosaic formation of the ganglion cell subtype marked by SPIG1 expression. On the other hand, the glutamate-mediated afferent activity seems not to be required for the elimination of neighboring cells, because ribbon synapses have not been established at this stage [56]–[58]. The cell loss between P6 and P12 occurs position-independently (Figure 8B), implying that the increase of the exclusion zone between P6 and P12 is mainly due to the retinal growth.

In conclusion, we postulate that lateral inhibition of the cell fate decision and/or repulsive interaction during the lateral migration are the major factors responsible for the formation of the regular mosaics for the MTN-projecting RGCs, which are already observed at P1. After that spatially selective death of neighboring homotypic cells between P3 and P6 contributes to the completion of the mosaic. The latter is probably induced by an activity-dependent mechanism after the establishment of dendritic stratification on the cholinergic strata at P3, which roughly corresponds to the peak period of cell death (P2–P5).

### Direction-selective retinal circuit

Elucidating how a direction-selective system arises in the visual system is a general aim of systems neuroscience [25]. We suggest that SPIG1-positive cells in the pan-ventronasal retinal domain correspond to one of the three functional subtypes of ON DSGCs, which prefer a specific direction. Here, the ON DSGC subtype which prefers temporal to nasal direction is suggested to project to the DTN [27]. Therefore, the SPIG1-positive cells projecting to the MTN are presumably the upward or downward selective GCs. Another type of RGC, which encodes motion direction in the mouse retina, is the ON-OFF DSGC projecting to the dLGN and SC [70], [71].

Analysis of this RGC type has shown that spatially asymmetric connections between DSGC subtypes and a selective subset of cholinergic amacrine cells underlie the directional selectivity in DSGCs [24]. However, nothing has been known about how functional subtypes of DSGCs are generated and the asymmetric connections are established during development. Our results demonstrated for the first time that subtypes of ON DSGCs are heterogenous in gene expression well before eye opening and visual responsiveness and that a subtype marked by SPIG1 stratifies close to the processes of cholinergic amacrine cells as early as P3 (Figure 4D–F).

### Mouse retina comprises two distinct domains with respect to *SPIG1* expression

**Figure 9:**
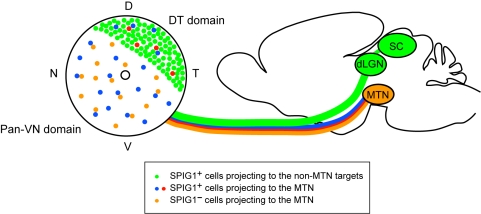
Schematic drawings of *SPIG1* expression profiles in the RGCs and their projection targets. In the DT domain, a large number of RGCs express *SPIG1* (green cells), and most of them project to the SC, dLGN, and AOS nuclei. In this domain, all MTN-projecting cells are marked by *SPIG1* expression (blue and red cells in the DT domain). In the pan-VN domain, GFP (SPIG1) is expressed exclusively in the MTN-projecting cells during the neonatal period (blue cells in the pan-VN domain). The MTN-projecting cells in this domain are divided into two distinct but interdependent regular mosaics, depending on the presence (blue cells) or absence (orange cells) of *SPIG1* expression. The cells of different populations (blue and orange cells) tend to exist as a pair. We suggest that the blue cells correspond to a functional subtype of the ON DSGC that prefers upward movement, whereas the orange and red cells correspond to a subtype that prefers downward movement.

Our results show that the mouse retina can be subdivided into two domains with respect to *SPIG1* expression along the dorsotemporal to ventronasal axis during development (Figure 9). In the dorsotemporal domain, *SPIG1* is expressed in RGCs for the retinothalamic/retinocollicular pathways mediating cortical vision and the AOS serving visuomotor function, but is not expressed in RGCs for the pretectal or hypothalamic pathway serving subcortical circadian functions or papillary light reflex. Characterization of morphological types of the *SPIG1*-expressing RGCs in the dorsotemporal domain is an issue for the future.

By P10, presumptive amacrine cell subsets start to express GFP, and at around eye opening (P13), the expression of GFP in RGCs begins to decrease in the pan-ventronasal domain (Figure S1, A–F). As a result, in adult retinas, GFP expression is detected not in RGCs but in choline acetyltransferase-negative amacrine cells with small, round cell bodies (<10 µm diameter), located in the GCL and INL (Figure S1, G–I, M–R). Thus, the cell-type-specific expression of *SPIG1* is developmentally regulated.

What is the meaning of the *SPIG1* expression domains? Our preliminary results indicate that the boundary dividing the two expression domains has already been established at the neonatal stage, and appears to remain in adults. It has been reported that the retina is comprised of genetically unique domains [30], [72]–[76]. The mouse retina is divided into dorsal and ventral fields by the uneven distribution of two spectral classes of cones, though their biological significance remains unclear [77]. Interestingly, NADPH-diaphorase-positive RGCs in the chick retina form a regular mosaic only in the dorsal retina [78]. *SPIG1* is expressed in a dorsotemporal-rich fashion also in the chick retina during development (our unpublished observation).

In this study, we demonstrated the usefulness of a molecular marker for a distinct type of RGC for studying development. Our results raise hopes that more molecular markers other than *SPIG1* may exist for distinct RGC types, especially in the topographically expressing molecules in the retina. Cell type-specific genes are presumably expressed in a unique pattern transiently during development or stably in the adult retina, because specific types of RGCs often distribute un-evenly in the retina. These genetic components may be involved in generating a type-specific dendritic morphology to exhibit the “tiling” phenomenon in the retina or might be important for generating specific types (and their specific functions) of RGCs. Knocking in the *SPIG1* gene in mice would be useful also for studying how motion-sensitive retinal circuits are established during development.

## Materials and Methods

### Generation of knock-in mice

A genomic fragment containing exon 2 of *SPIG1* was amplified from a BAC DNA clone by PCR using as primers 5′-GTCGACTGGCAGGCACTACTGGATGGCAGTGTGTTG-3′ (sense) and GTCGACCAACAAGACCACACCTCCTAATAGTGCCAC (antisense), and subcloned into a *Xho*I site of the pBluescript vector. The enhanced *gfp* (*gfp*) gene isolated from pEGFP-N1 (Clontech) was inserted into the signal sequence region in exon 2 of *SPIG1* gene, together with a phosphoglycerate kinase promoter-driven neomycin resistance gene (pGK-Neo): the first four amino acids, Met-Lys-Pro-Gly, of the signal peptide of SPIG1 were fused to the N-terminus of GFP via 14 amino-acids linker derived from the sequence of the multiple cloning site in pEGFP-N1. After homologous recombination, *gfp* should be expressed under control of the *SPIG1* gene regulatory unit. For negative selection, a diphtheria toxin A (DT-A) gene cassette was additionally placed at the 3′ end of the targeting construct. This construct was electroporated into R1 embryonic stem (ES) cells, and neomycin resistant cells were selected with G418. Genomic DNA prepared from the resistant clones was analyzed by PCR using TGAGATGGATGTGCGTGAGGGATTAAGCTC and CACCTTGATGCCGTTCTTCTGCTTGTCGGC, and by Southern analysis with a probe located in intron 1 (probe: PCR fragment amplified using TGCATCACGCTAACTTGTCATGAC and TTGGCACACTCTCCAAGAGAAATG). The evaluation of 144 independent clones revealed one that had been correctly targeted.

This ES cell line was introduced into eight-cell-stage C57BL/6J mouse embryos. Chimeras thus obtained were crossed with C57BL/6J mice to generate a heterozygous mutant (*SPIG1^gfp/+^*) through germline transmission. Genotyping of the progeny was performed by Southern blot analysis (as described above) or by PCR analysis of tail DNA using as primers GCTGGGATTAGAGATACAGACTGCCACTCC and GCACCAACTTCAGCAGCAGAAGTGCTGTGC. No *SPIG1* transcript was detected in the brain of *SPIG1^gfp/gfp^* mice by RT-PCR using GAGTCCGGCCACCTGGTCATTCCCTCG and GCAGTCCTTGCTGTGTACACTGACAAT. On the other hand, the *gfp* transcript was detected in the brain of *SPIG1^gfp/+^* and *SPIG1^gfp/gfp^* mice by RT-PCR using CAGCTGGACGGCGACGTAAAC and CACCTTGATGCCGTTCTTCTGC, indicating that GFP is expressed from the targeted allele. Confirmation that the targeting vector was not inserted into an ectopic genomic locus was obtained by Southern blot analysis with a probe for a coding region of *gfp*. The targeted mice were backcrossed for four generations to the C57BL/6J line. Experiments with animals were all carried out according to the guidelines of the National Institute for Basic Biology (Okazaki, Japan).

### Tissue preparation for histology

Mice were anesthetized with a ketamine/xylazine cocktail (ketamine, 28 ng/g body weight; xylazine, 3 ng/g body weight), and perfused intracardially with 4% paraformaldehyde (PFA) in PBS. For analyses of the retina, heads were decapitated, immersed in 4% PFA, and fixed overnight at 4°C. For analyses of the brain, they were dissected, immersed in 4% PFA, and fixed overnight at 4°C. Embryos were removed and fixed with 4% PFA overnight at 4°C.

For whole-mount immunohistochemistry, retinas were dissected and stored in PBS at 4°C until used for staining. For whole-mount *in situ* hybridization, retinas were rinsed in PBS/0.1% Tween-20 and dehydrated in an ascending series of graded methanol for 5 min each. Then the retinas were stored in 100% methanol at –20°C until used for experiments. For analyzing cross-sections of the retinas, heads including the eyes were infiltrated with a graded sucrose series to 18% (30% for brains), then embedded in OCT compound. The heads and brains were sectioned coronally and sagittally, respectively, and stored at –80°C until used for immunohistochemistry or *in situ* hybridization.

BrdU labeling of cells in the S phase of the cell cycle was performed as described previously [79]. BrdU (Sigma) was injected intraperitoneally into pregnant females at E15.5 (100 µg/g of body weight). The females were sacrificed 1 h after the injection, and the embryos were removed, fixed with 4% PFA, embedded in OCT compound, and sectioned at 16 µm.

### *In situ* hybridization

Section *in situ* hybridization was performed (section; 16 µm) as described previously [75]. Whole-mount *in situ* hybridization was performed as described previously [80]. The template used for the preparation of the digoxigenin (DIG)-labeled RNA probe was a 863-bp fragment of mouse *SPIG1* cDNA (nucleotide residues 975–1837; Genbank accession number AF374459) and a 795-bp fragment of *gfp* cDNA (2629–3423; U55762). Positive cells were stained purple with nitroblue tetrazolium salt and 5-bromo-4-chloro-3-indolyl phosphate toludinium salt. For sections of embryonic retinas and neonatal brains, *in situ* hybridization signals were visualized by a tyramide amplification signal detection system using the DAKO GenPoint system (DAKO K0620), according to the manufacturer's instructions. Finally, positive cells were stained brown with diaminobenzidine, and brain sections were counterstained with toluidine blue. Images were acquired with an optical microscope (BX51; Olympus).

### Immunohistochemistry

Cholinergic cell processes were visualized with anti-VAChT antibody (Promega). Immunolabeling for both cross-sections (30 µm) and whole-mounts was carried out as follows: The retinal tissues were incubated with anti-VAChT antibody at a 1∶1000 dilution and/or anti-GFP antibody (nakalai tesque) at 1∶500 in 1% blocking reagent (Roche Diagnostics GmbH)/0.5% Triton-X in PBS (PBST) for 3–6 d at 4°C. The specimens were then washed with PBS at room temperature, and subsequently blocked with 1% blocking reagent/PBST for 2 h at room temperature. Finally, they were incubated with Alexa 546 donkey anti-goat and/or Alexa 488 donkey anti-rat IgG antibody (Invitrogen) at 1∶500 each in 1% blocking reagent/PBST for 2 h. After three rinses in PBS, nuclei were stained with TO-PRO-3 (Invitrogen) at 1 µM, mounted in Gelmount, and cover-slipped. Images were acquired using a laser-scanning confocal microscope (LSM510; Zeiss) with a 63x oil immersion objective (NA, 1.4; Zeiss) or a 40x water immersion objective (NA, 1.2; Zeiss). For whole-mounts, image stacks were collected along the z-axis at steps of 0.37-0.45 µm. For double staining of GFP and BrdU, sections were first processed for the GFP immunostaining. After that they were further fixed in 4% PFA for 15 minutes, washed three times in PBS, incubated in 0.5N HCl at 55°C for 6 minutes, washed three times in PBS, and stained with mouse anti-BrdU antibody (1∶100; Becton Dickson).

### Analyses of somatic diameter and dendritic stratification

For quantification of the somatic diameter, z-stack confocal images were taken through the GCL of flat-mount retinas: the somatic diameter was defined as the longest axis of the cell soma measured at 0.5 µm interval.

Stratification width and depth of dendrites in the IPL were determined in the retinal cross-sections (optical section; 3 µm). The boundaries of the IPL were visualized by labeling nuclei with TO-PRO-3. The location of fluorescence intensity along the IPL depth was determined, and plotted the distribution of the dendrites' location. The fluorescence intensity of dendrites along the IPL depth was analyzed using ImageJ (http://rsb.info.nih.gov/ij/). The location of the peak of the fluorescence intensity curve was used to determine stratification depth, whereas the width of the curve at half the maximum height (width at half maximum height; WHM) indicates the vertical spread of the dendrites within the IPL [38]. The stratification depth within the IPL was defined as 0–100% from the outer border (INL side) to the inner border (GCL side) of the IPL. Stratification levels were calculated individually for each GFP-positive soma. To determine the extent of overlap between red (processes of VAChT-positive cells) and green (dendrites of GFP-positive ganglion cells) profiles, we first calculated the WHM of the red and green peaks for each region. We then measured the overlap of the WHM of the red and green pixel intensity distributions and expressed this as a percentage of the WHM of the green profile.

### Anterograde labeling

RGC axons were anterogradely labeled with CTB conjugated to Alexa 555 (Invitrogen) as described previously [81]. In brief, mice were anesthetized, and their eyes were filled unilaterally with 0.5 µl of 0.5 mg/ml CTB-Axela 555 in PBS using a microinjector (IM-300; NARISHIGE) at postnatal day 0 (P0) or P9. After CTB injections, the mice were returned to home cages for a day, then reanesthetized, and perfused with 4% PFA. Their brains were dissected and sliced coronally at 100 µm with a vibratome. Labeled retinas were flat-mounted to confirm the complete labeling of the GCL. Some were unilaterally enucleated at P2. Images were acquired with the confocal microscope.

### Retrograde labeling

Somata and dendrites of RGCs were retrogradely labeled with CTB-Alexa 555 in the following way. Mice were anesthetized, and 100–200 nl of 0.5 mg/ml CTB-Alexa 555 was injected into the MTN or SC unilaterally. A glass pipette (tip diameter ∼30 µm) connected to a microinjector (IM-9B; NARISHIGE) was used for the tracer injection. The glass pipette was positioned on the stereotaxic head-holder (SG-4N; NARISHIGE) with an angle of 50° to avoid penetration of the microglass pipette into visual areas overlying the MTN as described previously [28]. A vertical approach was employed for the injection into the SC. After 24 h (for P0, P2, and P5 mice) or 48 h (for P10 mice), mice were perfused with 4% PFA. After postfixing, the brains were sliced at 100 µm using a vibratome and stained with TO-PRO-3 to locate the injection site. Retrogradely labeled retinas were removed and flat mounted, then the entire retina was photographed using a fluorescence microscope (Axiophoto2; Zeiss) with a 10x objective (NA, 0.3; Zeiss) to map all of the loci of labeled RGCs and GFP-positive cells in the GCL. Next, z-stack images of labeled RGCs at 0.45 µm steps were acquired to examine the colocalization of GFP and CTB-Alexa 555 signals using the confocal microscope with a 40x water immersion objective. The contrast of the final image was adjusted using Photoshop.

### Fluorescent *in situ* hybridization in conjunction with CTB labeling

We found that the Alexa dyes coupled to CTB maintain their fluorescence after prolonged heating inherent to the *in situ* hybridization procedure. Therefore, the retrograde tracing with CTB-Alexa was combined with the fluorescent *in situ* hybridization to detect mRNA within RGCs projectionally specified. One day after the injection of CTB-Alexa 488 unilaterally into the MTN, the mice were perfused with 4% PFA and retinal sections at 16 µm were prepared for fluorescent *in situ* hybridization. Brains were sliced at 100 µm with a vibratome and stained with TO-PRO-3 to locate the injection site. The hybridization protocol was the same as that for colorimetric *in situ* hybridization. Detection of the DIG-labeled probe was performed with alkaline phosphatase-conjugated anti-DIG antibody using a HNPP Fluorescence Detection kit (Roche Diagnostics GmbH). After sections were stained with TO-PRO-3, images were acquired using the confocal microscope with a 40x water immersion objective. At least six retinal sections were analyzed per animal.

### Analysis of the mosaic

We mapped and counted GFP-positive cells (endogenously SPIG1-positive cells), CTB-positive cells (retrogradely labeled cells from the MTN), and GFP·CTB-double-positive cells in the GCL in the flat-mount retina. The spatial organization of the labeled cells was investigated by obtaining the density recovery profile (DRP) as described previously [43]. The DRP describes the spatial autocorrelation within a single population of cells, or the crosscorrelation between two types of cells. For the DRP in the pan-ventronasal domain, three fields were sampled from the dorsonasal, ventronasal, and ventrotemporal quadrants (900×900 µm in the respective mid-peripheral region). For the DRP in the dorsotemporal domain, a similar field was sampled from the dorsotemporal quadrant. For the DRP, each cell in the field was considered as a reference cell and the distribution of distances of all other cells from the reference cell was plotted. A second cell was next treated as a reference cell, and a similar distribution measured. For the DRP of crosscorrelation between GFP^+^·CTB^+^ and GFP^−^·CTB^+^ cells, each GFP^+^·CTB^+^ cell was considered as a reference cell and the distribution of distances of all GFP^−^·CTB^+^ cells from the reference cell was plotted. When the results were binned, the DRP shows the average distribution of distances from any chosen cell. In this study, the cells were binned at 10 µm intervals. When the cells are randomly distributed, the profile becomes flat with a value equal to the mean spatial density. Increasing values towards a reference cell indicate a clustering distribution, and decreasing values indicate anti-clustering. Estimated DRP graphs based on isotropic retinal growth and random cell death were obtained as follows. First, the DRP graphs were stretched along the Y-axis based on a retinal increase in length. A factor of the retinal increase in length was calculated by extracting the square root of the increase in a whole retinal area. Second, the graphs were scaled down along the X-axis based on decreases in cell density accounted for by a retinal increase in area and a reduction of MTN-projecting cells in a whole retina, assuming that the cell loss occurs independently of the distance from a reference cell. Thus the bin sizes of the estimated graphs are more than 10 µm. The obtained graphs were fitted to the real data of the respective next developmental stage. The size of the exclusion zone in the autocorrelation was quantified using the effective radius (ER) as previously described [43]. In brief, the ER was obtained as the difference between (i) and (ii), where (i) is the shortest distance (D) for which the histogram of DRP reaches its average plateau density, and (ii) is the quotient of the integral of DRP between zero and D divided by the average plateau density. The ERs were analyzed using one-way ANOVA followed by a Fisher's PLSD post hoc test with StatView software.

## Supporting Information

Figure S1 GFP expression in the retina after eye opening. A-F, GFP expression in the RGCs projecting to the MTN and SC in the pan-ventronasal domain at P13. CTB-Alexa 555 was injected into the MTN (A–C) or SC (D–F) of *SPIG1^gfp/+^* mice at P11, and examined at P13. In addition to the MTN-projecting cells (CTB+), other neuronal populations with small cell bodies start to express GFP in the GCL (A–C). SC-projecting cells (CTB+) are devoid of GFP expression (D–F), suggesting that these GFP-positive cells with small cell bodies in the GCL are displaced amacrine cells. GFP-positive cells are also found in the INL (data not shown). G–L, GFP expression in the RGCs projecting to the MTN in the adult retina. CTB-Alexa 555 was injected into the MTN of *SPIG1^gfp/+^* mice at P60, and examined four days later. In the pan-ventronasal domain, MTN-projecting cells are devoid of GFP expression (G–I). In contrast, in the dorsotemporal domain, all of the MTN-projecting cells are marked by GFP (J–L). M–R, Double staining with anti-choline acetyltransferase (ChAT) antibody (1∶100; Chemicon) and anti-GFP antibody for the pan-ventronasal domain of the adult retina. ChAT is a marker for cholinergic amacrine cells. In the INL (M–O) and GCL (P–R), ChAT-negative amacrine subsets with small, round cell bodies (<10 µm) are GFP-labeled. The immunostaining protocol for anti-ChAT antibody was the same as that for anti-VAChT antibody. Scale bars: 100 µm (C, F, I, L); 50 µm (O, R).
(7.66 MB EPS)

Figure S2 DRP analysis for the MTN-projecting cells in each retinal quadrant at P6. A–D, DRPs for CTB^+^ cells (MTN-projecting cells) in dt (A), dn (B), vt (C), and vn (D) retinal quadrants. CTB^+^ cells show no clear exclusion zones (n = 3). The density of CTB^+^ cells in the dt quadrant (A) is apparently lower than that in other quadrants (B–D). E–H, DRPs for GFP^+^·CTB^+^ cells. DRP in the dt quadrant (E) shows that these cells do not form a regular mosaic (n = 3). Note that in the dt quadrant, the GFP^+^·CTB^+^ cell population (E) is identical to the CTB^+^ cell population (A) because all CTB-labeled cells are GFP-positive in this quadrant. The density of GFP^+^·CTB^+^ cells in the dt quadrant (E) is accordingly higher than that in other quadrants (F–H). In contrast, DRPs in other quadrants (F–H) show that these cells form a regular mosaic in the GCL with an exclusion zone (approximately ∼50 µm). Values are the mean±SE.
(0.73 MB EPS)

Figure S3 DRP analysis for the GFP^+^ cells in the pan-ventronasal domain at E17.5. The DRP for GFP^+^ cells shows peaks at around 20–40 µm from the reference cell, indicating a sign of clustering (3 fields from 1 retina). The retina increased in area by approximately two-fold between E17.5 and P1, whereas the overall population density decreased by approximately half during the same period (see Figure 8B). Values are the mean±SE.
(0.64 MB EPS)
